# Effects of Deep Versus Moderate Neuromuscular Blockade in Laparoscopic Gynecologic Surgery on Postoperative Pain and Surgical Conditions: Protocol for a Randomized Controlled Trial

**DOI:** 10.2196/resprot.9277

**Published:** 2018-07-09

**Authors:** Edoardo De Robertis, Anna Caprino Miceli, Giorgio L Colombo, Antonio Corcione, Yigal Leykin, Luigia Scudeller, Enrico Vizza, Paolo Scollo, Società Italiana Ginecologia E Ostetricia, Società Italiana Di Anestesia Analgesia Rianimazione E Terapia Intensiva

**Affiliations:** ^1^ Department of Neurosciences, Reproductive and Odontostomatological Sciences Section of Anaesthesia and Intensive Care Medicine University Federico II Naples Italy; ^2^ Anestesia e Rianimazione Cannizzaro Hospital Catania Italy; ^3^ Department of Drug Sciences School of Pharmacy University of Pavia, Italy Milan Italy; ^4^ Studi Analisi Valutazioni Economiche Milan Italy; ^5^ Thoracic Surgery AORN dei Colli Vincenzo Monaldi Hospital Napoli Italy; ^6^ Anesthesia and Intensive Care Azienda per l'Assistenza Sanitaria n. 5 - Friuli Occidentale Pordenone Italy; ^7^ Scientific Direction Clinical Epidemiology Unit IRCCS San Matteo Foundation Pavia Italy; ^8^ Gynecologic Oncology, Regina Elena Hospital Rome Italy; ^9^ Maternity and Early Childhood Department Gynecology and Obstetrics Cannizzaro Hospital Catania Italy

**Keywords:** clinical trial, laparoscopic surgery, anaesthesia, neuromuscular blockade

## Abstract

**Background:**

Postoperative pain, especially shoulder pain, is commonly reported after laparoscopic gynecologic procedures. Some studies suggest that a lower insufflation pressure may reduce the risk of postoperative pain; however, there is no agreement on the optimal pneumoperitoneum pressure during gynecologic laparoscopic surgery or whether lower pressure would lead to clinically significant improvements without increasing operative complications. Questions remain regarding the clinical significance of improvements, safety, and cost-effectiveness of deep neuromuscular blockade with low-pressure pneumoperitoneum.

**Objective:**

The primary objective of this study was to assess the superiority of anesthesia with deep neuromuscular blockade with pneumoperitoneum 8 mm Hg over moderate blockade with pneumoperitoneum 12 mm Hg in terms of overall pain 24 hours after surgery in adult women undergoing pelvic surgery for hysterectomy or benign adnexal diseases. Effects on the intensity and timing of postoperative pain in specific locations, surgeon satisfaction, respiratory and hemodynamic stability, operating times, and direct and indirect costs will be assessed.

**Methods:**

In this multicenter, randomized controlled trial with a superiority design, 300 patients will be randomly allocated in the ratio 1:1 to moderate neuromuscular blockade with a target insufflation pressure of 12 mm Hg or deep neuromuscular blockade with a target insufflation pressure of 8 mm Hg, with stratification by type of surgery and clinical center. The patient, the statistician, and the nurse who will assess the primary endpoint will be blinded to the allocation.

**Results:**

Recruitment to this trial is expected to open in June 2018 and is expected to close in June 2019.

**Conclusions:**

This study is designed to confirm the reported benefits of postoperative pain and provide additional data needed to address questions regarding the effects of this intervention on operating theater management and direct and indirect costs. Strengths of this protocol include the large sample size distributed among diverse institutions across the Italian territory and the collection and analysis of data on numerous secondary objectives. Limitations include the possible introduction of bias because the surgeon and anesthesiologist are not blinded to the intervention.

**Registered Report Identifier:**

RR1-10.2196/9277

## Introduction

Postoperative pain, especially shoulder pain, is commonly reported after laparoscopic gynecologic procedures [1]. Some studies have suggested that using a lower insufflation pressure may reduce the risk of postoperative pain [2]; however, there is no agreement on the optimal pneumoperitoneum pressure during gynecologic laparoscopic surgery or whether lower pressure would lead to clinically significant improvements without increasing operative complications. In a study of 100 laparoscopic cholecystectomy procedures randomly allocated to low-pressure pneumoperitoneum (8 mm Hg) or normal pressure (14 mm Hg), low pressure significantly decreased the frequency and intensity of postoperative shoulder pain, analgesics consumption, and length of hospital stay [2].

In gynecologic procedures, a study of 150 patients undergoing gynecologic laparoscopy randomly allocated to abdominal insufflation pressures of 8 mm Hg (n=54), 12 mm Hg (n=45), or 15 mm Hg (n=51), the pain scores were found to be significantly better with low insufflation pressure; however, there was a trend toward longer operation times and increased hemorrhage in this group [3]. A recent systematic review did not confirm this reported increase in operating time or blood loss with lower pressure; however, it did raise questions regarding whether the benefit of the observed reduction in postoperative pain could offset the decrease in the quality of surgical conditions [4]. The authors reported an association between lower pressure and increased risk of poorer surgical field visibility (relative risk 10.31; 95% CI 1.29-82.38).

Thus, there appears to be a consensus that low insufflation pressures can reduce postoperative pain [5-9], and this would appear to suggest a way to improve surgical conditions at lower insufflation pressures. For this purpose, it has been suggested that neuromuscular blockade (NMB) may help to maintain a sufficient intra-abdominal workspace at lower insufflation pressure [10]. NMB induces dose-dependent muscle relaxation that allows the muscles to stretch to their maximum length [11]. This may improve the surgical working space during laparoscopic procedures.

The advantages of deep NMB in laparoscopic surgery are not well established. Several studies have examined the effect of deep NMB on the working surgical space and the relationship between relaxation and insufflation pressure in nongynecologic [12-14] and gynecologic procedures [15,16]. The use of deep NMB compared with moderate NMB maintained during surgery is associated with improved surgical conditions during laparoscopic procedures (reviewed in [17]). NMB also improved surgical conditions when suturing the abdominal fascia [18]. A randomized controlled trial comparing deep NMB with 8 mm Hg pneumoperitoneum with moderate NMB with 12 mm Hg pneumoperitoneum in laparoscopic hysterectomy revealed a reduction in postoperative shoulder pain, with no differences in duration of surgery, length of hospital stay, or time to recovery of daily activities [19]. Thus, questions remain regarding the clinical significance of improvements, safety, and cost-effectiveness of deep NMB with low-pressure pneumoperitoneum.

Economic efficiency should not be considered reductively, in terms of mere cost savings, but in its most correct sense by determining the best anesthetic conditions for the patient and the surgeon and by identifying the best relationship between efficacy, safety, and cost-effectiveness in the use of health resources [20]. Optimal management of anesthesia, including the type of NMB, can facilitate the conduction of surgery and have positive effects on peri- and postoperative outcomes. New NMB agents and their antagonists allow precise control of awakening times for efficient operating room scheduling, avoiding cost increases because of personnel overtime. For example, rapid recovery may prevent some of the frequent and numerous side effects associated with longer sedation and the associated increases in hospital cost [21].

The availability of neuromuscular monitoring and agents to reverse the effects of nondepolarizing muscle relaxants has made the use of deep NMB safer and more practical. Reversal agents work either by increasing acetylcholine levels through competitive inhibition of acetylcholinesterase (neostigmine) or by encapsulating aminosteroid NMB agents (sugammadex) [22]. Neostigmine is associated with a higher risk of postoperative residual curarization, compared with sugammadex [23,24]. Sugammadex rapidly forms an essentially irreversible dose-dependent chelating complex with rocuronium and other aminosteroid muscle relaxants [25]. This allows reversal of all levels of NMB with complete recovery of muscle function almost immediately after administration.

The purpose of this study was to investigate whether deep NMB with reduced pressure pneumoperitoneum is superior to moderate NMB with normal pressure pneumoperitoneum, in terms of overall pain 24 hours after waking in patients undergoing gynecologic laparoscopic procedures. In addition, the effects on the intensity and timing of postoperative pain in specific locations, surgeon satisfaction, respiratory and hemodynamic stability, operating times, and direct and indirect costs will be assessed.

## Methods

### Study Objectives

The primary objective of this study was to assess the superiority of anesthesia with deep NMB with pneumoperitoneum 8 mm Hg over moderate blockade with pneumoperitoneum 12 mm Hg, in terms of overall pain 24 hours after waking in adult women undergoing pelvic surgery for hysterectomy or benign adnexal diseases.

Secondary objectives will be to assess differences between the 2 groups in terms of:

- Patient relaxation quality during surgery
- Need for administration of additional NMB agents based on intraoperative train-of-four (TOF)
- Surgeon satisfaction (Likert scale: 1=impossible to proceed, 2=insufficient, 3=sufficient, 4=good, and 5=excellent) on each of the following:
  - neck strain
  - back strain
  - visual acuity
  - overall satisfaction
- Hemodynamic stability during surgery, determined clinically by monitoring systolic and diastolic blood pressures, electrocardiogram and, in part, by capnography.
- Respiratory stability during surgery, determined clinically by monitoring ventilation parameters: O_2_ saturation, end-tidal CO_2_, and CO_2_ insufflation pressure; tidal volume; positive end-expiratory pressure; inspiratory-to-expiratory ratio; fractional O_2_ percentage; and blood gases.
- Direct and indirect costs
- Duration of surgery (from first access to umbilical closure, in minutes)
- Time to awakening (from induction to awakening, in minutes)
- Time from the end of the surgery to awakening (modified Wilson sedation scale)
- Time to discharge from postanesthesia care unit (PACU; from induction to discharge, in minutes)
- Time in operating theater stay from entry to discharge to PACU or ward
- Postoperative nausea and vomiting (PONV), at the same time points as for pain, using a 0 to 10 numerical rating scale (NRS)
- Time to discharge, evaluated with the Postanaesthetic Discharge Scoring System [26]

### Trial Design

This will be a multicenter, randomized controlled trial with a superiority design. Patients will be randomly allocated in the ratio 1:1 to one of two parallel groups with stratification by type of surgery and clinical center.

### Eligibility Criteria for Participants

Eligible women scheduled for an elective laparoscopic or robotic gynecologic procedure with an expected duration <90 min performed under general anesthesia and requiring tracheal intubation (eg, cystectomy, hysterectomy, salpingo-oophorectomy) will need to satisfy the following criteria:

- Age 18-60 years
- Body mass index between 20 and 30 kg/m^2^
- American Society of Anesthesiologists class 1 or 2
- Able to provide informed consent to trial procedures (eg, no speech or hearing impairment or language barriers)

Patients meeting any of the following criteria will be excluded:

- Pregnancy
- Surgery for endometriosis, diagnostic laparoscopy with chromosalpingoscopy, myomectomy, or tube ligation
- Anticipated airway difficulty
- Requirement for rapid sequence induction
- Anticipated intensive care unit admission or when extubation is not planned
- Hepatic or renal failure
- Baseline heart rate <50 bpm
- Documented or suspected neuromuscular disorders, Guillain-Barré syndrome, cerebrovascular accidents with residual neurologic deficits, Parkinson disease, and myasthenia gravis
- Patients receiving fusidic acid or toremifene 24 hours before surgery and hormonal contraceptives
- Patients receiving drugs for or affected by medical conditions that may prolong or shorten the duration of rocuronium effect (eg, aminoglycosides, magnesium)
- Patients with a history of allergy to rocuronium, neostigmine, or sugammadex
- Any condition making the administration of patient satisfaction questionnaire difficult or impossible.

### Setting and Data Collection Locations

Participating centers will be selected among those where laparoscopic and/or robotic gynecologic surgery is standard of care, performing at least 100 laparoscopic hysterectomies per year (or 200 laparoscopic procedures per year), with sampling distributed geographically across Italy. Both university and general hospitals can participate. At each center, a single surgeon (and team) and a single anesthesiologist (and team) will be involved.

### Intervention/Treatment

#### Preparation for Surgery and Induction of Anesthesia

All patients will undergo intestinal preparation on the day preceding surgery and antibiotic prophylaxis (according to local hospital guidelines) 30 min before skin incision.

Premedication, for example, with midazolam 0.04 mg/kg, desametasone 0.1 mg/kg, and H2 antagonists will be performed before anesthesia induction in reception (actual drugs will be selected by the anesthesiologist).

Intraoperative monitoring will include electrocardiography, noninvasive arterial pressure measurements, nasogastric tube placement, and pulse oximetry. In addition, acceleromyography (using a dedicated instrument) will be used to monitor the response of the adductor pollicis muscle. Neuromuscular monitoring and management will follow Good Clinical Research Practice guidelines.

Anesthesia will be induced with propofol 2-2.5 mg/kg, remifentanil 0.1 µg/kg/min, and desflurane 4% as standard dosages, using target-controlled infusers.

Before rocuronium administration, the acceleromyography instrument will be calibrated and stabilized; a 50-Hz tetanic stimulation will be applied for 5 s, the acceleromyography instrument will be calibrated, and a series of TOF measurements will be documented for >2 min until a stable baseline is obtained (<5% variation in the TOF ratios).

Trendelenburg position will be maintained as required for surgery.

Analgesic transition will be achieved with fentanest 100 gamma at induction. Starting 20 min before the end of surgery, postoperative analgesia will be ketorolac 90 mg/24 hours in continuous infusion.

#### Group A (Intervention): Deep Neuromuscular Blockade

Patients in group A will undergo anesthesia with deep NMB attained during surgery using rocuronium 0.6 mg/kg, followed by orotracheal intubation within 60 s to 120 s after confirmation of relaxation and intra-abdominal CO_2_ insufflation to a pressure of 8 mm Hg.

Anesthesia will be maintained with target-controlled infusion of propofol and remifentanil while monitoring the bispectral index (A-2000 BIS monitor; Aspect Medical Systems, Inc, Natick, MA, USA). After induction, rocuronium will be continuously infused and titrated to maintain the posttetanic count (PTC) at 1 to 2 throughout surgery. NMB will be reversed at the end of surgery with sugammadex 4 mg/kg at PTC of 1 or 2.

#### Group B (Control): Moderate Neuromuscular Blockade

Patients in group B will undergo anesthesia with moderate NMB with rocuronium bromide 0.6 mg/kg, followed by orotracheal intubation within 60 s to 120 s after confirmation of relaxation and intra-abdominal CO_2_ insufflation pressure of 12 mm Hg.

Anesthesia will be maintained with target-controlled infusion of propofol and remifentanil while monitoring the bispectral index (A-2000 BIS monitor; Aspect Medical Systems Inc). After induction, rocuronium will be continuously infused and titrated to maintain TOF response at 1-2 throughout surgery. NMB will be reversed at the end of surgery with sugammadex 2 mg/kg.

### Insufflation Pressure

The target insufflation pressure will be different in the 2 groups. However, the intraperitoneal pressure will be adjusted to the lowest pressure necessary to maintain the surgical field. The lowest stable (ie, preserving a viable surgical field) intraperitoneal pressure reached during surgery will be recorded.

### Reversal and Predischarge Procedures (Including Postoperative Nausea and Vomiting Prophylaxis)

Patients in group A will have NMB reversed with intravenous sugammadex at 4 mg/kg at PTC of 1 or 2 and those in group B with sugammadex 2 mg/kg at a TOF count of 1 or 2. The time from administration of the reversal agents to a TOF ratio of 0.9 will be recorded (time to reversal).

Analgesic transition will be continued (as described previously). PONV prophylaxis will be achieved with coinfusion of 8 mg intravenous ondansetron+100 mg intravenous ranitidine, and the patient will be awakened in the operating theater.

Table 1 provides a comparison between intervention and control groups.

**Table 1:** Comparison of intervention and control groups.

Intervention	Group A (intervention): deep neuromuscular blockade	Group B (control): moderate neuromuscular blockade
Type of NMB^a^	Deep	Moderate
Drug	Rocuronium bromure 0.6 mg/kg	Rocuronium bromure 0.6 mg/kg at induction
Orotracheal intubation	Within 60-120 s	Within 60-120 s
Intra-abdominal insufflations	8 mm Hg	12 mm Hg
NMB maintenance	Target-controlled infusion of propofol and remifentanil while monitoring the bispectral index; rocuronium will be continuously infused and titrated to maintain the PTC^b^ at 1-2 throughout surgery	Target-controlled infusion of propofol and remifentanil while monitoring the bispectral index; rocuronium will be continuously infused and titrated to maintain TOF^c^ response at 1-2 throughout surgery
NMB reversal	Sugammadex 4 mg/kg at PTC of 1 or 2	Sugammadex 2 mg/kg

^a^NMB: neuromuscular blockade.

^b^PTC: posttetanic count.

^c^TOF: train-of-four.

**Table 2:** Secondary outcomes.

Outcome	Definition/variable	Measurement
Pain	Rescue dose needed, yes/no	Determined clinically
Pain	Rescue doses in the first 24 and 48 hours, n	Determined clinically
Pain	AUC^a^ of NRS^b^ at specific sites: intrascapular, incisional, lower abdomen	NRS at predefined time points plus at rescue dose request
Pain	Maximum pain, Time to maximum pain, Time to pain <4	NRS at predefined time points plus at rescue dose request
Patient movement	Any patient movement	Reported by the surgeon or anesthesiologist
Patient movement requiring recurarization during surgery	Any patient movement	Reported by the surgeon or anesthesiologist
Surgeon satisfaction	Likert scale 1-5; neck strain, back strain, visual acuity, overall satisfaction	Questionnaire: every 15 min from first laparoscopic view until removal of laparoscopes at the end of surgery or up to 8 hours from the first score.
Hemodynamic stability during surgery	Yes/no	Determined clinically: systolic and diastolic blood pressures, total diuresis, need for catecholamines, etc
Respiratory stability during surgery	Yes/no	Determined clinically by monitoring PaO_2_, PaCO_2_, etc
Duration of surgery from first access to umbilical incision closure	Measured in minutes	N/A^c^
Time from administration of NMB^d^ antagonist to awakening	Measured in minutes	N/A
PONV^e^	AUC of NRS 0-10 in the first 24 hours	N/A
Time from surgery to discharge	Measured in days	Postanesthesia Discharge Scoring: ≥2 assessments > 8.5 hours apart
Postoperative evaluation	Composite endpoint	Telephone questionnaire [26]
Direct costs	Number, description, and type of drug; type of intervention (International Classification of Diseases, Ninth Revision, ICD9 code); year; operating theater time; NMB reversal time (end of surgery to extubation); type and number of personnel present; anesthesia; presence/absence of postoperative residual curarization (PORC); prophylactic therapy for PORC; rescue therapy for PORC; PACU^f^ stay, yes/no; PACU stay duration, minutes; PACU stay >60 min, yes/no; intensive care unit admission, yes/no; with immediate extubation?, with early extubation?, with delayed extubation?	These data will be registered on a dedicated monitoring form for each patient.
Indirect costs	Absence from work; lost productivity; QoL^g^	EuroQoL-5 dimensions questionnaire (possibly other appropriate instruments)

^a^AUC: area under the curve.

^b^NRS: numerical rating scale.

^c^N/A: not applicable.

^d^NMB: neuromuscular blockade.

^e^PONV: postoperative nausea and vomiting.

^f^PACU: postanesthesia care unit.

^g^QoL: quality of life.

### Outcome Measurements

#### Primary Outcome

The primary outcome will be the area under the curve (AUC) of overall pain in the first 24 hours after surgery, assessed on an NRS of 0 to 10 administered by a nurse blinded to study group allocation.

AUC for overall pain will be calculated on the NRS measurements at predetermined time points (30 min, 60 min, 120 min, 4 hours, 8 hours, 12 hours, and 24 hours) plus any time when rescue analgesic is requested.

#### Secondary Outcomes

Secondary outcomes are summarized in Table 2.

Procedures lasting >90 min from first access to umbilical incision closure will be excluded from the analysis. The surgeon will rate surgical conditions every 10 min during the procedure and again at the end, using a 5-point scale (1=excellent, 2=good, 3=acceptable, 4=poor, and 5=inadequate).

In the economic analysis, direct costs will be based on operating and patient recovery times in the context of each hospital center, including time in the operating room and PACU and costs for the professionals monitoring the patient before extubation and resumption of spontaneous respiration. Indirect costs will be captured with the EuroQOL-5 dimensions quality of life questionnaire, lost productivity, and absence from work.

### Sample Size Calculation

A sample size determination was conducted for the main outcome variable. Hypothesizing an AUC for overall pain in the first 24 hours of 144 patients in group B and 120 patients in group A, a common SD of 60, 140 patients per group will be necessary to achieve 92% power with an alpha error of 5% with a 2-sided *t* test for independent samples.

On the basis of preliminary results of a pilot study conducted at the Catania center, we expect to lose no more than 5% of patients because of surgery durations >90 min; thus, we plan to enroll 300 patients to be allocated in the ratio 1:1 in 2 groups.

### Interim Analysis

No interim analysis is planned.

### Randomization: Sequence Generation, Allocation Concealment Mechanism, and Implementation

#### Sequence Generation

Allocation sequence to groups A and B will be obtained using the “ralloc” module in Stata 14 (StataCorp LLC, College Station, Texas), with blocks of variable size (4-6-8), and stratified by participating center and type of surgery (hysterectomy). The algorithm for sequence generation will be maintained by the study statistician and will not be communicated to any additional study staff.

#### Allocation Concealment Mechanism

The study statistician will prepare the appropriate number of numbered, opaque, sealed envelopes for each center. These will be maintained at each center by an appropriately trained research nurse. At the time of anesthesia induction, the surgical nurse will open the relevant envelope, and the anesthesiologist will proceed to the allocated treatment.

### Data Collection and Management

Study data on the primary and secondary outcomes will be collected from clinical charts and by dedicated data personnel using Research Electronic Data Capture electronic data capture tools [27]. The electronic database will be built by bioinformatics experts and will include built-in quality checks for key variables.

### Statistical Analysis

#### Statistical Methods for Analyzing Primary and Secondary Outcomes

The primary endpoint (AUC for pain) will be compared by Student *t* test. *P* value <.05 will be considered significant. Descriptive statistics will be obtained for all variables assessed in the study population. Mean and SD will be used for normally distributed variables, and mean and interquartile range will be used for skewed distributions and proportions for categorical variables. Whenever relevant, 95% CIs will be calculated.

For group comparisons, Student *t* test (rank-sum test or Mann–Whitney test for skewed distributions) will be used for quantitative variables (analysis of variance or Kruskal-Wallis for >2 groups, respectively) and Pearson chi-square test (Fisher exact test where appropriate) for categorical variables. Two-tailed tests will be used in all cases. *P* value <.05 will be considered significant.

Generalized mixed models will be used to assess differences between groups in endpoints measured at several time points.

A detailed statistical analysis plan will be developed after the first 40 patients have been enrolled.

#### Methods for Any Additional Analyses

The main analysis will be on the whole study population. Additional analyses will be stratified by type of intervention. Also, analyses will consider uterus volume as a proxy for complexity of surgery.

#### Blinding

Anesthesiologist and surgeon will be aware of the treatment assignment. The patient, the nurse who will assess the primary outcome (NRS on day 1), and the statistician will be blinded.

### Ethical Issues

This protocol, patient information sheet, and patient consent form have been reviewed and approved by the local ethics committee of Udine. All participating centers will obtain approval from their local ethics committee before starting enrollment. Italian law requires that approval is obtained first from the ethical committee of the coordinating center before obtaining approval from the other participating centers. Any protocol modifications will be submitted for review by each ethical committee. The study has been registered at Udine Ethical Committee (registration number: 23445/Ceur—4/9/2017). Written informed consent will be obtained directly from each patient by the participating anesthesiologist at the presurgery visit. Italian law does not allow consent from health care surrogates.

## Results

Recruitment to this trial is expected to open in June 2018 and is expected to close in June 2019.

## Discussion

Laparoscopic surgery provides benefits that include less bleeding, faster recovery, and shorter hospital stays. However, it is performed in a restricted space that may limit the surgeon’s view and range of motion. Higher CO_2_ insufflation pressure provides more insufflation volume [28] and improves surgical field visibility [29] but is associated with increased postoperative side effects [1]. During laparoscopic procedures, maintaining deep NMB, compared with moderate NMB, is associated with improved surgical conditions, as reviewed in Madsen and colleagues’ study [17]. NMB also improved surgical conditions when suturing the abdominal fascia [18]. However, the advantages of deep NMB in laparoscopic surgery are not well established, and questions remain regarding the clinical significance of the improvements, safety, and cost-effectiveness.

This study may confirm the reported benefits for postoperative pain and provide the additional data needed to address questions regarding the effects of this intervention on operating theater management and direct and indirect costs.

Strengths of this protocol include the large sample size and the participants being distributed among diverse institutions across the Italian territory, which will increase the generalizability of the results. The study will provide a comprehensive picture of the effect of and the collection and analysis of data on numerous secondary objectives, including an analysis of direct and indirect costs, to determine the overall effect of the intervention. Limitations include the possible introduction of bias because the surgeon and anesthesiologist will not be blinded to the intervention. However, potential bias will be reduced through blinding of the nurse who will assess the primary outcome (ie, the AUC of overall pain during the first 24 hours after surgery measured on an NRS of 0-10). Moreover, the randomization sequence will be generated centrally and allocation concealed in opaque, sealed envelopes until the time of anesthesia induction.

## Abbreviations

AUC: area under the curve
NMB: neuromuscular blockade
NRS: numerical rating scale
PACU: postanesthesia care unit
PONV: postoperative nausea and vomiting
PTC: posttetanic count
TOF: train-of-four
